# X Chromosome-Specific Repeats in Non-Domestic Bovidae

**DOI:** 10.3390/genes15020159

**Published:** 2024-01-25

**Authors:** Svatava Kubickova, Olga Kopecna, Halina Cernohorska, Jiri Rubes, Miluse Vozdova

**Affiliations:** Department of Genetics and Reproductive Biotechnologies, Central European Institute of Technology-Veterinary Research Institute, Hudcova 70, 621 00 Brno, Czech Republic; svatava.kubickova@vri.cz (S.K.); halina.cernohorska@vri.cz (H.C.); jiri.rubes@vri.cz (J.R.)

**Keywords:** Bovidae, FISH, laser microdissection, repetitive sequence, sequence analysis, qPCR, X chromosome

## Abstract

Repetitive sequences form a substantial and still enigmatic part of the mammalian genome. We isolated repetitive DNA blocks of the X chromosomes of three species of the family Bovidae: *Kobus defassa* (KDEXr sequence), *Bos taurus* (BTAXr sequence) and *Antilope cervicapra* (ACEXr sequence). The copy numbers of the isolated sequences were assessed using qPCR, and their chromosomal localisations were analysed using FISH in ten bovid tribes and in outgroup species. Besides their localisation on the X chromosome, their presence was also revealed on the Y chromosome and autosomes in several species. The KDEXr sequence abundant in most Bovidae species also occurs in distant taxa (Perissodactyla and Carnivora) and seems to be evolutionarily older than BTAXr and ACEXr. The ACEXr sequence, visible only in several Antilopini species using FISH, is probably the youngest, and arised in an ancestor common to Bovidae and Cervidae. All three repetitive sequences analysed in this study are interspersed among gene-rich regions on the X chromosomes, apparently preventing the crossing-over in their close vicinity. This study demonstrates that repetitive sequences on the X chromosomes have undergone a fast evolution, and their variation among related species can be beneficial for evolutionary studies.

## 1. Introduction

A substantial part of eukaryotic genomes comprises repetitive sequences, which can be classified as interspersed elements or tandem arrays. The interspersed elements widely distributed throughout the genome are mainly represented by transposable elements (TEs) while the tandemly arrayed sequences include multigene families, such as ribosomal RNAs (rRNA) and histone genes, satellite DNA and micro- and minisatellites [1]. Apart from highly repeated sequences, low copy number sequences and low repeated DNAs reside in the eukaryotic genome.

Contrary to theories that have been widely accepted in the past, there is mounting evidence that repetitive sequences represent transcriptionally active regions [2]. The most abundant transposable element within the human genome is represented by long interspersed nucleotide elements (LINEs). The only active lineage of LINEs found within humans belongs to the LINE1 class. Most of LINE1s, which include more than 500 thousand copies, are not active in the genome as they are truncated repeats. Only the full-length copies are capable of self-copying. The presence of the LINE1 element at a certain locus can affect gene expression and even lead to the formation of alternative transcripts. This can make a significant contribution to the functions of individual cells, tissues and the whole organism [3]. The transcriptional activity of a satellite DNA depends on its location. The level of transcripts detectable within the active human centromere is low. This contrasts with the higher transcriptional levels of pericentromeric satellites, which are necessary for heterochromatin maintenance [4]. Some simple repeated sequences are elements known to be transcribed in the context of repeat expansion disorders [5].

It was shown that satellite DNA transcripts have an important role in the epigenetic processes of chromatin formation [6,7], and are indispensable for the structural and functional organization of the genome [8,9]. Heterochromatic regions are known to harbour active genes. Modifications in these regions might give rise to fertility barriers that promote evolutionary divergence and speciation [1,10,11]. Furthermore, the transformation of euchromatin into heterochromatin has been recognised as a relevant factor in speciation processes [12,13].

In mammals, the repetitive DNA sequences are located mainly in centromeric chromosome regions. Moreover, various classes of repetitive DNA accumulated in non-recombining regions of sex chromosomes during their evolution that most probably proceeded independently of autosomes [14]. The X chromosomes of species included in the family Bovidae analysed in this study diversified through chromosomal rearrangements, embracing inversions, centromere shift, heterochromatic variation and translocations with autosomes [15,16,17,18,19,20,21,22]. Therefore, the bovid X chromosomes show high variation among subfamilies, tribes and even between genera belonging to one tribe [17,23,24,25]. There are three main types of X chromosomes within the family Bovidae: one submetacentric in morphology represented by the tribe Bovini and two acrocentric referred to as the “eland-type” (tribe Tragelaphini) and the “suni-type” (Caprini, Cephalophini, Hippotragini, Alcelaphini, Antilopini, Aepycerotini and Peleini) [16]. In contrast, the evolution of bovid Y chromosomes was driven mainly by the accumulation of repetitive DNA that was accompanied by Robertsonian fusions with autosomes in several species of Antilopini and Tragelaphini [23,24,25].

Little is known about the composition and distribution of repetitive sequences present in the sex chromosomes of bovid species. Most studies have focused on highly repetitive sat DNA resident in centromeric or pericentromeric regions [26,27,28]. The intercalary X- or X/Y-specific repetitive sequences were described and characterized only in *Capra hircus*, *Ovis aries*, *B. taurus* and *Bubalus bubalis* [29] and in several Antilopini species [24,25]. Cabelova et al. [30] found repetitive DNA sequences specific to the Y chromosomes of 11 species representative of nine tribes of the family Bovidae.

The determination of the accurate localization and composition of repetitive sequences remains problematic despite current easily available sequencing techniques. Even completely sequenced genomes may have multiple gaps in the centromeric and other regions occupied by repetitive DNA sequences. Sex chromosomes are particularly difficult to assemble because they contain a high amount of repetitive sequences [31,32]. In Bovidae, the complete sequence of the Y chromosome of the *C. hircus* species, with the genome ‘sequenced to completion’, still remains unavailable. Additional studies of repeated segments are required for a better understanding of the genome structure and function. Therefore, integrating DNA sequence data with a chromosomal mapping of the repeated elements by means of cytogenetic techniques can provide a more comprehensive picture, even in completely sequenced genomes [1].

Microdissection techniques represent an alternative approach in the isolation of repetitive DNA sequences of interest that enable their further analyses by sequencing and mapping. Laser microdissection was used, for instance, by [31] to assess the genetic content of the W chromosome of the flour moth (*Ephestia kuehniella*). This approach allowed us to determine families of transposable elements, microsatellites and recent mitochondrial DNA insertion sites. In Bovidae, this technique was used for the isolation of repeats present in centromeric regions [23,24,27,28,29,33] and in the Y chromosome [30,34].

In this study, we employed laser microdissection for the isolation and characterization of three repetitive sequences present in the X chromosomes of bovid species *K. defassa*, *B. taurus* and *Antilope cervicapra*. We studied their distribution in a wide range of species of the family Bovidae and in three outgroup species representative of the families Cervidae, Giraffidae and Antilocapridae.

## 2. Materials and Methods

### 2.1. Chromosome Preparation

Material from 41 bovid species and 3 outgroup species used in this study was analysed (see Table 1, Table 2 and Table 3). Blood samples of 38 taxa were collected from captive-born animals held in the Zoological Gardens in Dvur Kralove nad Labem, Prague, Plzen, Liberec and Olomouc (Czech Republic) by experienced zoo veterinarians on the occasion of preventive and diagnostic examinations during the years 2003–2015, and an aliquot of the blood was used for our studies. The blood cells were cultured according to standard protocols. Fibroblast cell cultures from *O. oreotragus* and *A. americana*, and cell suspensions of *R. sharpei* were obtained from the Evolutionary Genomics Group, Department of Botany and Zoology, University of Stellenbosch, South Africa. Cell cultures were grown and harvested using conventional procedures [35]. Metaphase spreads were prepared using standard cytogenetic techniques. For the identification of the chromosomal regions of interest, C- or G-banding was performed as previously described [16]. The systematic classification used in this study followed Groves and Grub (2011) [36]. The study complies with the current laws of the Czech Republic and the Republic of South Africa. All institutional and national guidelines for the care and use of animals were followed.

### 2.2. Isolation of X Chromosome Repetitive DNA Sequences

C-banding positive regions of the X chromosomes of *A. cervicapra* were microdissected by the MicroLaser system (Carl Zeiss MicroImaging GmbH, Munich, Germany). The pooled DNA was amplified using DOP-PCR (degenerate oligonucleotide primed polymerase chain reaction) without pretreatment as previously described [37]. Amplification products were cloned into a pDrive vector (Qiagen, Hilden, Germany). Species-specific clones were selected using DOT BLOT hybridization [29], fluorescently labelled and checked for specificity by FISH. Plasmid DNA was subsequently isolated and sequenced. Sequences comprised satellite DNA but were not long enough to represent the whole basic repeat unit obtained by PCR (BRU-PCR). Therefore, primers amplifying the 5′- and 3′-flanking regions were designed. A simplified version of inverse PCR was performed on isolated untreated genomic DNA. The primers were chosen with the emphasis on the distance between primers being as short as possible (see Supplementary Table S1). This permitted the retrieval of almost the full-length DNA basic repeat unit [24]. Amplification products were cloned and sequenced (ACEXr sequence).

Primers for inverse PCR were designed (see Supplementary Table S1) and inverse PCR was performed on genomic DNA of *K. defassa*. Amplification products were cloned and sequenced (KDEXr sequence).

The BTAXr clone involving the BRU of *B. taurus* was constructed on the basis of the sequence of the bovine X centromeric-specific repeat (NCBI accession number AJ884576) published by Pauciullo et al. [29]. Primers were selected from this clone for inverse PCR but no amplification product was obtained using bovine genomic DNA as a template. Therefore, we searched in NCBI for sequences similar to the clone AJ884576 using BLASTN searches. Several regions with a high similarity to the sequence NW_001501982 were found. We analysed this 330 kb sequence and revealed the 1999 bp motif present in multiple copies but not arranged in tandem. This 1999 bp sequence was amplified from bovine DNA by PCR (for primers see Supplementary Table S1), cloned and sequenced (BTAXr sequence).

The sequences of our clones were compared with those in the NCBI database using BLASTN searches. BLAST2 was used to assess sequence homologies. DNA sequences were screened for interspersed repeats by RepeatMasker (http://www.repeatmasker.org).

The plasmid clones containing the basic repeat unit were labelled with biotin-16-dUTP (Roche, Mannheim, Germany) using the BioPrime^®^ Array CGH Genomic Labeling Module (Invitrogen, Carlsbad, CA, USA) and used for FISH analysis.

### 2.3. Fluorescence In Situ Hybridization

Metaphase spreads for FISH (fluorescence in situ hybridization) analysis were prepared from lymphocytes or fibroblast cell cultures using standard methanol:acetic acid (3:1) fixation. Fluorescent probes were hybridized as previously described [27]. Slides were washed in 0.4 × SSC/0.3% Igepal at 73 °C, counterstained and mounted with DAPI/Antifade. The FISH preparations were evaluated using Olympus BX 51 and BX 60 microscopes equipped with the necessary fluorescence filters and automated pad shifts. Good quality metaphase cells were scanned using a CCD camera and evaluated using image analysis (ISIS 3, MetaSystems, Altlussheim, Germany).

### 2.4. Detection of X Chromosome Repetitive DNA Sequences Using PCR

We probed for the presence of X chromosome repetitive DNA sequences in species representative of bovid tribes that gave negative results after FISH (see Table 2). Genomic DNA was extracted from blood samples using QIAamp DNA Blood Mini Kit (Qiagen, Hilden, Germany). Primers were designed according to the sequences of KDEXr, BTAXr and ACEXr clones (see Supplementary Table S1) to produce 1 kb, 2 kb and 1 kb PCR products, respectively. Cycling parameters were 95 °C for 4 min for initial denaturation, 30 cycles at 95 °C for 1 min, 61 °C (KDEXr, ACEXr) or 58 °C (BTAXr) for 1 min and 72 °C for 2 min, with a 5 min final extension at 72 °C. The amplification products were visualized on a 2% agarose gel to prove the presence of the specific sequence in the species of interest. Amplification products obtained from several representative species were sequenced, and the sequences were compared using BLAST2.

### 2.5. Analysis of X Chromosome Repetitive DNA Sequences Using Quantitative Real-Time PCR

Genomic DNA was isolated using the QIAamp DNA Blood Mini Kit, and the concentration was measured using NanoDrop Lite Spectrophotometer (Thermo Fisher Scientific, Waltham, MA, USA). Quantitative real-time PCR (qPCR) was carried out in iQ 96-Well PCR Plates using the LightCycler^®^ 480 System (Roche, Mannheim, Germany). For absolute quantification analysis, serial dilutions of an external standard with a known concentration were used to create a standard curve. Additionally, 1 kb, 2 kb and 1 kb amplification products were prepared from KDEXr, BTAXr and ACEXr recombinant clones, and used as standards. Each PCR plate comprised the internal calibration curve referring to the clone of interest. All qPCR reactions were performed in duplicates and contained 1× SYTO-9 Master Mix (Top–Bio, Prague, Czech Republic), 1.6 μM primers and 10–35 ng of genomic DNA. PCR parameters for 40 cycles were the same as described above for PCR detection. Fluorescence data were acquired on SYBR Green I / HRM Dye-detection format at the end of each 72 °C extension step of the PCR. Crossing point (Cp) values from samples and calibration curves were processed using LightCycler^®^ 480 quantification software and absolute amounts of the examined repetitive sequence were obtained. These absolute values were loaded to a Copy number calculator (http://scienceprimer.com, accessed on 31 July 2020) to determine copy numbers of the distinct repetitive sequences present in genomic DNA of one cell. The bovine haploid DNA content comprising 2,857,605,192 bp [38] was used for all investigated species.

### 2.6. Detection of BTAXr Sequences on Microdissected Chromosomes

The presence of BTAXr sequences was confirmed separately on X chromosomes and autosomes of *A. imberbis* and *A. americana* using PCR. X chromosomes and autosomes from the same metaphase cell were collected using laser microdissection into two different tubes. Ten cells were microdissected for two rounds of PCR. Primers 5′-CAAAGAATAAAGGGCACTGAAGG-3′ and 5′-GATTGGTCAGCTAGGCACTGC-3′ were selected according to the BTAXr sequence to amplify the 200 bp product. The amplification products from the second PCR were analysed using gel electrophoresis on a 2% agarose gel.

## 3. Results and Discussion

### 3.1. KDEXr Clone

#### 3.1.1. Sequence Analysis

The KDEX clone from *K. defassa* was sequenced and the sequence data representing the BRU-PCR were deposited in the NCBI database under accession number KP677335. The BRU-PCR length was 3465 bp. The screening of the KDEX sequence using RepeatMasker revealed the presence of interspersed repeats comprising 44% of its total length (LINE1: 29%, DNA transposons hobo-Activator-Tam3 (hAT): 15%). LINEs are transposable elements, widespread among eukaryotes. The LINE1 identified here was represented by inactive truncated repeats. According to several studies, LINE1 transcripts are implicated in the epigenetic regulation of numerous genes in normal embryonic development and also in tumorigenesis [39,40,41]. In our study, to prevent potential unspecific hybridization in subsequent FISH analyses, the 1 kb segment of the KDEX sequence without interspersed repeats was amplified, labelled and termed KDEXr (primers for the KDEXr sequence are listed in Supplementary Table S1. The same segment was amplified from the genomic DNA of *R. fulvorufula*, *A. melampus*, *C. elaphus*, *A. americana* and sequenced (Supplementary Table S2). A comparison of these sequences using BLAST2 revealed sequence similarities varying from 75% (*C. elaphus* vs. *R. fulvorufula*) to 93% (*A. americana* vs. *A. melampus*). BLASTN searches found high similarity (74–90%) for the KDEXr sequence with sequences present on the X chromosomes of *C. hircus* (six matches in two loci), *O. aries* (nine matches in two loci), *B. taurus* (eight matches in one locus) and also on autosomes of *C. hircus* (CHI12), *O. aries* (OAR7) and *B. taurus* (BTA17, BTA22). Additionally, the KDEXr sequence occurs with 74–78% similarity on four sites of the Y chromosome of *B. taurus*. BLASTN searches revealed the presence of the KDEXr sequence orthologue even in more phylogenetically distant species—*S. scrofa* (70% similarity), *V. pacos* (68% similarity), *A. melanoleuca* (67% similarity) and *C. s. simum* (66% similarity)—indicating an ancestral origin of this sequence (for taxonomic classification see Table 1).

#### 3.1.2. FISH Analysis

**Figure 1:**
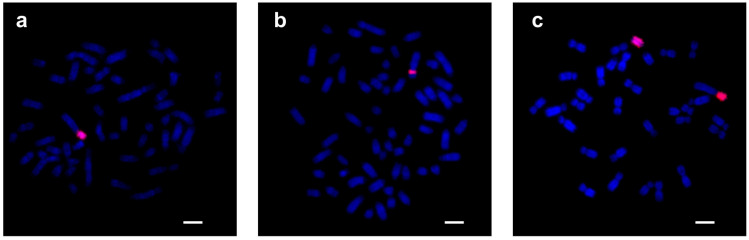
FISH results with KDEXr, BTAXr and ACEXr probes (red). (**a**) FISH with the KDEXr probe on chromosomes of *K. defassa*. (**b**) FISH with the BTAXr probe on chromosomes of *B. taurus*. (**c**) FISH with the ACEXr probe on chromosomes of *A. cervicapra*. Chromosomes were counterstained with DAPI (blue). Scale bar represents 5 µm.

The KDEXr clone generated from *K. defassa* was fluorescently labelled and used as a probe for the FISH analysis of various species of the family Bovidae and three representatives of other families (Cervidae, Giraffidae and Antilocapridae). The results of these hybridization experiments are summarized in Table 2 and Table 3. The distribution of KDEXr sequences in *K. defassa* is shown in Figure 1a.

**Figure 2:**
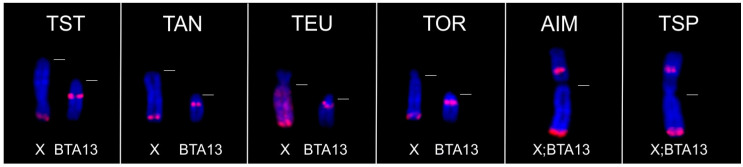
FISH with the KDEXr probe (red) to chromosomes X and BTA13 of Tragelaphini species: *T. strepsiceros* (TST), *T. angasii* (TAN), *T. eurycerus* (TEU), *T. oryx* (TOR), *A. imberbis* (AIM) and *T. spekii* (TSP). The dashed white lines indicate the position of centromeres.

The KDEXr probe hybridized successfully to the X chromosomes of almost all representatives of the family Bovidae with the exception of three species (*A. melampus*, *A. marsupialis*, *M. kirkii*) which showed no FISH signal. Interesting FISH patterns were seen in species belonging to the tribe Tragelaphini (see Figure 2), which is characterized by the fusion Y;BTA13 [23].

**Figure 3:**
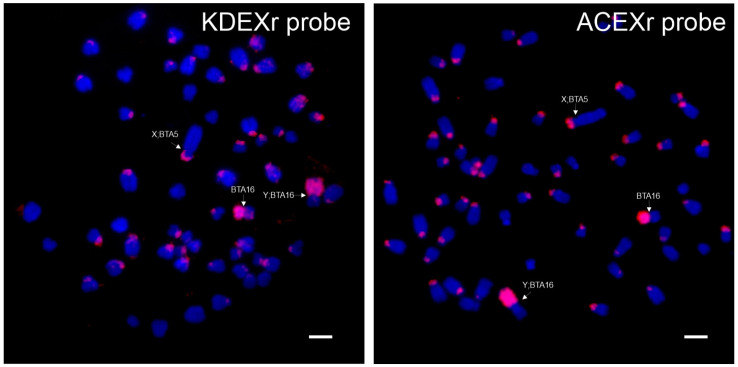
FISH with KDEXr and ACEXr probes (red) to chromosomes of *E. thomsonii*. Scale bar represents 5 µm.

Two species (*A. imberbis*, *T. spekii*) also showing X-autosomal fusion X;BTA13 exhibited FISH signals not only on the distal part of the X chromosome, but also on the proximal part of BTA13 (at the site of the fusion of both chromosomes). Similarly, in the Tragelaphini species with the unfused X chromosome, strong hybridization signals were seen both on the X chromosome (distal part) and on unfused chromosome BTA13 (pericentromeric region). Different FISH patterns showed two Antilopini species (*N. dama*, *E. thomsonii*) which are distinguished from other Antilopini by the fusion Y;BTA16. In both species, a strong fluorescent signal of the KDEXr probe covered the entire Y chromosome. Additionally, in *N. dama*, weaker signals occurred in centromeric regions of three meta/submetacentric autosomes. *E. thomsonii*, on the contrary, exhibited fluorescent signals on p arms of all autosomes, including the X chromosome (see Figure 3). Small p arms characteristic of this species also comprise sat II DNA [25] and seem to be completely heterochromatic.

**Figure 4:**
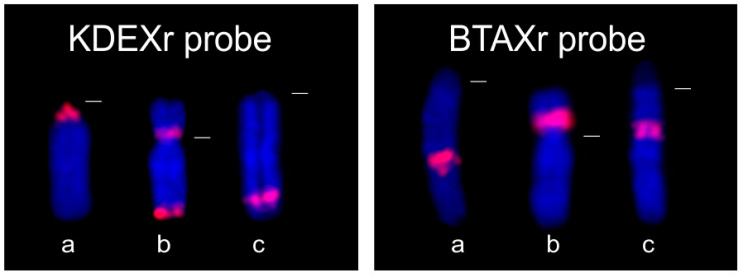
FISH with KDEXr and BTAXr probes (red) to X chromosomes of (**a**) *C. hircus* representing the caprine type of the X chromosome, (**b**) *B. taurus* representing the bovine type of the X chromosome, (**c**) *B. bubalis* representing the eland type of the X chromosome. The dashed white lines indicate the position of centromeres.

The physical distribution of KDEXr sequences on bovid X chromosomes presented three main patterns (see Figure 4). The localizations in the pericentromeric region or on Xp arms were characteristic of species belonging to the subfamily Antilopinae (pericentromeric—Caprini, Hippotragini, *Oreotragus*, Alcelaphini, Reduncini; Xp—Antilopini, Alcelaphini, Reduncini). On the contrary, all species representative of the subfamily Bovinae manifested the FISH signals of the KDEXr probe on the distal part of Xq. This dissimilar distribution of the KDEXr sequence in both groups is linked to the dissimilarities between their X chromosomes caused by the reshuffling of several segments of the bovine type of the X chromosome during the evolution of Antilopinae species. These rearrangements had resulted in the formation of a caprine (suni) type of the X chromosome where the pericentromeric part Xq11–Xq12 (comprising our KDEXr sequences) corresponds to the distal part (Xq34–Xq41) of the bovine type of the X [15,19,42,43].

Our initial FISH experiments with the KDEXr probe on *K. defassa* had implied that the KDEXr clone might be exclusively X-specific. However, FISH experiments with the KDEXr probe showed that this clone can occupy both sex chromosomes. In Bovidae, Cabelova et al. [30] reported repeats specific to the Y chromosome in 37 species representative of the family Bovidae. Repetitive sequences specific to both sex chromosomes were previously found in reindeer [44,45] and muntjac [46], both being ruminant species from the family Cervidae. This phenomenon was described not only in ruminants but also in plants [47], insects [48,49], fish [50] and birds [51], supporting the idea of the independent evolution of sex chromosomes [44].

Concerning our FISH results, it seems that the predominant presence of large blocks of the KDEXr repetitive sequence on the X chromosome might denote its initial occurrence on this chromosome. In several species, the shuffling of the KDEXr sequence between X and Y chromosomes might arise during meiosis at some point during karyotypic evolution. In the meiosis of several Tragelaphini species, chromosomes X, Y;BTA13 and unfused BTA13 orthologue form a trivalent where the proximal parts of the X and the unfused BTA13 occur in close proximity [52]. This configuration probably enabled the shifting of the KDEXr sequence from the X chromosome to BTA13 via recombination during evolution. Some repetitive sequences found in genomes, such as LINEs, may occur on multiple chromosomes. For example, the LINE1 sequence can be localised at centromeric and noncentromeric positions on both autosomes and sex chromosomes. The link between LINE1s and inter- and intrachromosomal rearrangements, as well as a link between LINE1s and structural functions at centromeres, has recently been confirmed [53]. It is believed that on the X chromosome, the nonrandom organization of LINEs might be responsible for its facultative heterochromatisation. The LINE1 composition on the human X chromosome is fundamentally distinct from that of human autosomes. These nonrandom properties of LINE1 distribution on the X chromosome may serve as DNA signals to propagate inactivation along the X chromosome [54].

The results of our BLASTN searches and a wide occurrence of the KDEXr sequence across the family Bovidae suggest an ancestral origin of this sequence. To verify this assumption, we analysed three species representative of phylogenetically older ruminant families—*C. elaphus* (Cervidae), *G. camelopardalis* (Giraffidae) and *A. americana* (Antilocapridae). For FISH experiments in these species, the part of the KDEXr clone comprising no interspersed repeats was fluorescently labelled and used as a probe. We obtained positive FISH results (see Table 3) only in *C. elaphus*, which is phylogenetically closer to Bovidae than *G. camelopardalis* and *A. americana* [55,56].

#### 3.1.3. Analysis of KDEXr Sequence Using PCR and qPCR

According to our previous experience [24], we decided to use PCR for the detection of the KDEXr sequence in species, which gave negative results when using FISH. We assumed that the KDEXr sequence might be present in their genome but dispersed or in an insufficient amount to be visualised when using FISH. For PCR and the subsequent qPCR detection of the KDEXr sequence, primers amplifying the region without interspersed repeats were used.

We obtained positive PCR amplification both in bovid species—*A. marsupialis*, *A. melampus*, *M. kirkii*—and two outgroup species—*C. elaphus*, *A. americana*. Contrary to *C. elaphus*, the band of *A. americana* on the gel was very weak, indicating a potential low copy number of the KDEXr sequence in this species. Our findings were subsequently confirmed by qPCR which revealed a presence of 700 copies of the KDEXr sequence in the genomic DNA of *C. elaphus* in contrast to only 2 copies present in *A. americana* (see Table 4). The detection by qPCR also verified the low copy numbers of the KDEXr sequence in *A. marsupialis*, *A. melampus* and *M. kirkii*, ranging from units to tens. On the contrary, hundreds to thousands of copies were detected in species demonstrating strong FISH signals.

In *G. camelopardalis*, the PCR analysis with *K. defassa* primers was negative, which can denote the absolute absence of the KDEXr sequence in the giraffe’s genome. However, this also might have been caused by a limited complementarity between the primers and the target even at the presence of the sequence in the giraffe genome.

Otherwise, our FISH analysis supplemented with PCR detection confirmed the presence of the KDEXr sequence in all investigated species belonging to the family Bovidae and in two species representative of the families Cervidae and Antilocapridae. In some species, the sequence was maintained in only a few copies, while it was amplified to thousands in several species. BLASTN searches revealed its occurrence not only within Artiodactyla but also within Perissodactyla and Carnivora, with the composition changed moderately during the evolution. Similarly, a highly repetitive DNA common to species of the family Cervidae and *B. taurus* was previously analysed [57]. O’Meally et al. [58] even found repetitive sequences shared between birds (*Gallus gallus*) and snakes (*Stegonotus cucullatus*, *Notechis scutatus*).

### 3.2. BTAXr Clone

#### 3.2.1. Sequence Analysis

The BTAXr clone from *B. taurus* was sequenced and the sequence data representing the BRU-PCR were deposited in GenBank under accession number KP677336. The BRU-PCR was 1999 bp in length. The screening of the BTAXr sequence by RepeatMasker revealed the presence of interspersed repeats comprising 20% of its total length (LTR elements—ERV class I: 15%). Endogenous retroviruses (ERVs) are present in the genome of all vertebrates and seem to be beneficial to their host [59]. In cattle, more than 13,000 ERVs were detected [60].

The BTAXr sequence was amplified from the genomic DNA of *N. dama*, *A. melampus*, *A. imberbis*, *H. equinus*, *C. elaphus*, *G. camelopardalis* and *A. americana* using the same primers as in *B. taurus*, and sequenced (Supplementary Table S2). The obtained sequences were compared using BLAST2, resulting in sequence similarities varying from 81% (*B. taurus* vs. *A. americana*) to 94% (*N. dama* vs. *H. equinus*). BLASTN searches showed a high similarity (82–89%) of the BTAXr sequence with sequences present on the X chromosomes of *B. taurus* (thirteen matches in two loci), *C. hircus* (five matches in two loci) and *O. aries* (five matches in two loci). We also found a high similarity (82–89%) of approximately one half of the BTAXr sequence with sequences located on BTA8, BTA9, BTA19 (*B. taurus*) and CHI15 (*C. hircus*).

#### 3.2.2. FISH Analysis

The BTAXr clone generated from *B. taurus* was fluorescently labelled and used as a probe in FISH experiments in various species of the family Bovidae and in three representatives of the outgroup families (Cervidae, Giraffidae and Antilocapridae). The results of these FISH analyses are presented in Table 2 and Table 3. The distribution of BTAXr sequences in *B. taurus* is shown in Figure 1b.

The BTAXr probe hybridized successfully only to species representative of two bovid tribes—Bovini and Caprini. In Bovini, FISH with the BTAXr probe gave positive results on the X chromosomes of all investigated species. Moreover, the genera *Bubalus* and *Syncerus* also showed signals on their Y chromosomes. In Caprini, the BTAXr probe gave a strong fluorescent signal on the X chromosomes of species belonging to the genera *Ovis*, *Capra* and *Ammotragus*. Autosomes and Y chromosomes showed no hybridization. Surprisingly, the genus *Ovibos*, represented by *O. moschatus*, provided no FISH signal. The FISH experiments in the outgroup species were all negative.

The physical distribution of the BTAXr sequences on the X chromosomes of Caprini species is different from that of the Bovini species (see Figure 4). Similar to the KDEXr sequences, the BTAXr sequences remained localized in one segment, which was shifted during the karyotype evolution of the bovid species. In Caprini with the caprine type of X chromosome, BTAXr sequences occupy approximately the region Xq32-Xq34, which corresponds to Xp12 of the bovine type of X (seen in *Bos* and *Bison*) and Xq23 of the eland type of X where we could see FISH signals in *Bubalus* and *Syncerus* [42,43]. In *C. hircus*, *O. aries*, *B. taurus* and *B. bubalis*, we obtained the same FISH patterns as Pauciullo et al. [29].

The results of our FISH analyses were inconsistent with the phylogeny of Bovidae. FISH confirmed the presence of BTAXr sequences only in Caprini and Bovini, which are phylogenetically distant tribes within Bovidae [55,56]. However, the invariable location of BTAXr sequences in species of both tribes indicates that the sequence might have arisen in a common ancestor and, during the speciation of Bovidae, it was amplified only in some species. Therefore, we decided to use PCR and qPCR for the detection of the BTAXr sequence in FISH-negative species where we supposed its possible rare occurrence.

#### 3.2.3. Analysis of BTAXr Sequence Using PCR and qPCR

PCR and the subsequent qPCR detection of the BTAXr sequence was performed in species representative of all bovid tribes and also on three outgroup species. This analysis showed that the BTAXr sequence is present in the genomes of all examined species, but mostly in very low copy numbers (see Table 4) that are insufficient to be visualised by FISH. The presence of BTAXr sequences in all three outgroup species (*C. elaphus*, *G. camelopardalis* and *A. americana*) indicates that the sequence had obviously arisen from an ancestor of Pecora. However, the deletion of isolated sequences in other tribes could also be the result of purifying selection [61].

According to our FISH results, it seemed that the occurrence of the BTAXr sequence is confined entirely to the sex chromosomes. To verify this, we decided to microdissect separately the X chromosomes and autosomes of *A. imberbis* and *A. americana*, species with easily distinguishable X chromosomes. Subsequent PCR analysis performed on both groups of chromosomes showed that the sequence is also present in autosomes of both species. We suppose that, similarly to the KDEXr sequence, BTAXr sequences preferentially occupy the sex chromosomes but can also be localized in low copy numbers in the rest of the genome.

Two species with high copy numbers of the BTAXr sequence (*A. cervicapra*—172, *A. americana*—114) unexpectedly showed no FISH signal. This can be caused by the presence of a higher number of copies on autosomes. The second explanation, which we prefer, could be that BTAXr sequences are localized mainly on the X chromosome but in a dispersed form that is below the detection limit of the FISH method.

### 3.3. ACEXr Clone

#### 3.3.1. Sequence Analysis

The ACEX clone from *A. cervicapra* was sequenced and the sequence data representing the BRU-PCR were deposited in GenBank under accession number KP677337. The BRU-PCR length was 4143 bp.

A shorter segment of the ACEX sequence, 1 kb in length (fragment 3039–4108 bp of KP677337), termed ACEXr was amplified from the genomic DNA of *M. kirkii*, *A. imberbis*, *B. bubalis* and *C. elaphus*, and sequenced (Supplementary Table S2). Only 27 bp simple repeat was revealed in the ACEX using RepeatMasker, and also the ACEXr sequence represents a repeat without any known specific internal elements. Primers for the ACEXr sequence are listed in Supplementary Table S1. The comparison of these sequences using BLAST2 revealed sequence similarities varying from 77% (*M. kirkii* vs. *A. imberbis*) to 91% (*A. imberbis* vs. *B. bubalis*). BLASTN searches showed a high similarity (82–89%) for the ACEXr sequence with sequences present on the X chromosomes of *B. taurus* (three matches in one locus), *C. hircus* (three matches in two loci) and *O. aries* (three matches in two loci). Additionally, an 80% similarity with sequences of chromosomes Y and BTA22 of *B. taurus* was found.

#### 3.3.2. FISH Analysis

The ACEXr clone generated from *A. cervicapra* was fluorescently labelled and used as a probe for FISH analysis in species representative of ten bovid tribes and in one outgroup species (*C. elaphus*). The results of these hybridization experiments are summarized in Table 2 and Table 3. The distribution of ACEXr sequences in *A. cervicapra* is shown in Figure 1c.

Contrary to both clones described above, FISH experiments with the ACEXr probe gave positive results in only five species belonging to the tribe Antilopini. Even within this tribe, two species (*A. marsupialis* and *M. kirkii*) showed no fluorescent signal. The hybridization motifs were not uniform in FISH positive species. In *A. cervicapra* and *G. leptoceros* (both characterized by the fusion X;BTA5), a strong signal of the ACEXr probe covered the whole Xp arms and also the whole Y chromosome. Fluorescent signals present on the Y chromosome formed a block interrupted in the middle by sat I in *G. leptoceros* [24]. In *N. dama*, which has the Y;BTA16 fusion, the ACEXr probe hybridized to the whole sexual part of the Y chromosome and weakly to centromeres of two pairs of autosomes. In *E. thomsonii*, the hybridization with the ACEXr probe resulted in strong fluorescent signals covering almost the whole sexual part of Xq and the whole sexual part of the Y chromosome (see Figure 3). Moreover, ACEXr sequences were found on p arms of most autosomes where they form an integral part of heterochromatin together with KDEXr sequences and satII [25]. The last FISH-positive species, *R. sharpei*, showed signals at the pericentromeric region of the X chromosome.

In this study, we obtained FISH motifs comparable to those of Cernohorska et al. [24]. Considering the fact that the ACEXr probe hybridized successfully only to Antilopini species, we assumed that the ACEXr sequence could have arisen from an ancestor of this tribe. Then it would be evolutionary the youngest in comparison with KDEXr and BTAXr. However, our previous experience led us to perform PCR and qPCR analysis to confirm the supposed absence of ACEXr sequences in the genomes of the studied species that were FISH negative.

#### 3.3.3. Analysis of the ACEXr Sequence Using PCR and qPCR

The PCR detection of the ACEXr sequence revealed its presence in the genomes of all examined bovid species representative of ten bovid tribes and also in the genomic DNA of one outgroup species (*C. elaphus*). No band on the gel was obtained while analysing *G. camelopardalis* and *A. americana*. Subsequent qPCR detection confirmed that the ACEXr sequence occurs in low copy numbers in almost all examined species except three Antilopini species (*N. dama*, *A. cervicapra*, *G. leptoceros*) where the sequence was amplified up to thousands (see Table 4). The copy numbers of the ACEXr sequence varying from tens to thousands in species of the same tribe suggest that its rapid amplification occurred after divergence from a common ancestor and is therefore species-specific. This attribute was also described in satellite sequences (reviewed in Plohl et al. [62]).

Our PCR analyses of three outgroup species showed that the ACEXr sequence is present in *C. elaphus* contrary to *G. camelopardalis* and *A. americana.* The families Antilocapridae and Giraffidae had diverged earlier than Bovidae and Cervidae [55,56,63]. Therefore, we suppose that the ACEXr sequence had probably arisen in an ancestor common to Bovidae and Cervidae, after the divergence of Giraffidae.

### 3.4. Co-Localization of KDEXr, BTAXr and ACEXr Sequences in the X Chromosomes of O. aries, C. hircus and B. taurus

BLASTN searches found several matches for all three studied clones on the X chromosomes of *O. aries* (NC_019484), *C. hircus* (NC_022322) and *B. taurus* (AC_000187). When we aligned all the matches on the X chromosome of distinct species, we obtained interesting patterns (see Supplementary Table S3). In all three species, the ACEXr sequence was present in one or two copies surrounded by higher amounts of KDEXr sequences. BTAXr sequences formed a ‘block’ interrupted in *O. aries* and *C. hircus* by one KDEXr sequence and joined by one ACEXr sequence. Deeper investigation of regions comprising our repetitive sequences showed that these repeats are interspersed among regions rich in genes. BLASTN searches did not find a tandem arrangement for any of our three sequences on NC_019484, NC_022322 and AC_000187. The low numbers of obtained matches do not correspond with strong signals after FISH with KDEXr and BTAXr probes that provide evidence of the presence of high amounts of copies.

## 4. Conclusions

In summary, we isolated and characterized three different repetitive sequences present in blocks on the X chromosomes of *K. defassa*, *B. taurus* and *A. cervicapra*. All three clones investigated in our study hybridized preferentially to the X chromosomes but could also be detected on the Y chromosomes and autosomes. According to the searches for homologies to our repeats in NCBI, it seems that all three repetitive sequences analyzed in this study are interspersed on the X chromosomes among regions rich in genes. This distribution can be connected with the role of repeated sequences in preventing the crossing-over in their close vicinity and thus keeping the integrity of the sex chromosome architecture. The PCR and qPCR analysis used for the detection and absolute quantification of the studied repetitive sequences revealed that they can be present even in species which gave FISH-negative results.

On the basis of the distribution of the analysed repetitive sequences within the family Bovidae and three outgroup species, we suppose that the KDEXr sequence is evolutionarily the oldest in comparison with BTAXr and ACEXr sequences. KDEXr sequence, although changed to a certain extent, also occurs in species from very distant taxa (Perissodactyla and Carnivora). On the contrary, the BTAXr sequence does not seem to occupy the genome of any non pecoran species. Our results indicate that it might have originated in an ancestor of Pecora, more recently than the KDEXr sequence. The ACEXr sequence, as probably the youngest, seems to arise from an ancestor common to Bovidae and Cervidae, after the divergence of Giraffidae.

## Figures and Tables

**Table 1:** Taxonomy of species analysed in this study.

Class	Order	Suborder	Infraorder	Family	Species
Mammalia	Carnivora			Ursidae	*Ailuropoda melanoleuca*
	Perissodactyla			Rhinocerotidae	*Ceratotherium simum simum*
	Artiodactyla	Tylopoda		Camelidae	*Vicugna pacos*
		Suina		Suidae	*Sus scrofa*
		Ruminantia	Pecora	Giraffidae	*Giraffa camelopardalis*
				Antilocapridae	*Antilocapra americana*
				Cervidae	*Cervus elaphus*
				Bovidae	see Table 2

**Table 2:** FISH results with KDEXr, BTAXr and ACEXr probes on species representing 10 bovid tribes.

Subfamily	Tribe	Species (Sex-Autosomal Fusion)	KDEXr Probe			BTAXr Probe			ACEXr Probe		
			X	Y	A	X	Y	A	X	Y	A
Antilopinae	Caprini	*Ovis aries*	+	0	0	+	0	0	0	0	0
		*Capra hircus*	+	0	0	+	0	0	0		0
		*Ammotragus lervia*	+	0	0	+	0	0			
		*Ovibos moschatus*	+	0	0	0	0	0			
	Alcelaphini	*Connochaetes taurinus*	+	0	0	0		0	0	0	0
		*Connochaetes gnou*	+	0	0						
		*Connochaetes albojubatus*	+	0	0						
		*Damaliscus phillipsi*	+		0	0		0	0		0
	Hippotragini	*Oryx dammah*	+	0	0						
		*Oryx leucoryx*	+	0	0	0	0	0	0	0	0
		*Oryx gazella*	+	0	0						
		*Hippotragus equinus*	+	0	0	0	0	0			
		*Hippotragus niger*	+	0	0	0	0	0	0	0	0
		*Addax nasomaculatus*	+	0	0						
	Reduncini	*Kobus defassa*	+	0	0	0	0	0	0	0	0
		*Kobus ellipsiprymnus*	+	0	0						
		*Kobus leche*	+	0	0						
		*Kobus megaceros*	+	0	0						
		*Redunca fulvorufula*	+	+	0	0		0	0		0
	Aepycerotini	*Aepyceros melampus*	0	0	0	0		0	0		0
	Antilopini	*Nanger dama* (X;BTA5) (Y;BTA16)	+	+	+				0	+	+
		*Antilope cervicapra* (X;BTA5)	+	+	0	0	0	0	+	+	0
		*Antidorcas marsupialis*	0	0	0				0	0	0
		*Gazella leptoceros* (X;BTA5)	+		0				+	+	0
		*Raphicerus sharpei*	+		0	0		0	+		0
		*Madoqua kirkii*	0	0	0	0		0	0	0	0
		*Eudorcas thomsonii* (X;BTA5) (Y;BTA16)	+	+	+	0	0	0	+	+	+
	Oreotragini	*Oreotragus oreotragus*	+		0	0		0	0		0
Bovinae	Bovini	*Bos taurus*	+	0	0	+	0	0	0	0	0
		*Bubalus depressicornis*	+	0	0	+	+	0			
		*Bubalus bubalis*	+	0	0	+		0	0		0
		*Bison bonasus*				+	0	0			
		*Syncerus cafer*	+	+	0	+	+	0			
		*Syncerus cafer nanus*	+	0	0	+	+	0	0		0
	Boselaphini	*Boselaphus tragocamelus*(X;BTA14) (Y;BTA14)	*+*	0	0	0	0	0	0	0	0
	Tragelaphini	*Tragelaphus strepsiceros* (Y;BTA13)	+	0	+	0	0	0	0	0	0
		*Ammelaphus imberbis* (X;BTA13) (Y;BTA13)	+	0	0	0	0	0			
		*Tragelaphus angasii* (Y;BTA13)	+	0	+	0	0	0	0	0	0
		*Tragelaphus spekii* (X;BTA13) (Y;BTA13)	+	0	0						
		*Tragelaphus eurycerus* (Y;BTA13)	+	0	+						
		*Taurotragus oryx* (Y;BTA13)	+	0	+	0	0	0			

A autosomes; (+) positive FISH signals; (0) apparent absence of FISH signals.

**Table 3:** FISH results with KDEXr, BTAXr and ACEXr probes on outgroup species.

Subfamily	Tribe	Species	FISH Results								
			KDEXr Probe			BTAXr Probe			ACEXr Probe		
			X	Y	A	X	Y	A	X	Y	A
Cervidae	Cervini	*C. elaphus*	+	+	0	0	0	0	0	0	0
Giraffidae		*G. camelopardalis*	0	0	0	0	0	0			
Antilocapridae		*A. americana*	0	0	0	0	0	0			

(+) positive FISH signals; (0) apparent absence of FISH signals.

**Table 4:** Copy numbers of KDEXr, BTAXr and ACEXr sequences present in single cell genomic DNA determined by qPCR.

Family, Tribe, Species Analyzed			Sex	KDEXr	BTAXr	ACEXr
Bovidae	Caprini	*O. aries*	*♂*		166	12
		*O. moschatus*	*♂*		14	
	Alcelaphini	*C. taurinus*	♀		18	6
	Hippotragini	*H. equinus*	*♂*		4	2
	Reduncini	*K. defassa*	*♂*	5052	10	8
	Aepycerotini	*A. melampus*	*♂*	2	4	2
	Antilopini	*N. dama*	♀	24		40
		*N. dama*	*♂*	164		328
		*A. cervicapra*	♀		172	2882
		*A. marsupialis*	*♂*	16	6	12
		*G. leptoceros*	*♂*			1846
		*M. kirkii*	*♂*	16	4	12
	Oreotragini	*O. oreotragus*	♀		2	30
	Bovini	*B. taurus*	♀	530	508	10
		*S. cafer*	♀			50
	Boselaphini	*B. tragocamelus*	*♂*		8	26
	Tragelaphini	*A. imberbis*	♀		6	12
Cervidae	Cervini	*C. elaphus*	*♂*	700	6	2
Giraffidae		*G. camelopardalis*	*♂*		6	
Antilocapridae		*A. americana*	♀	2	114	0

## Data Availability

Data presented in this study are available in the article and Supplementary Material. The repetitive DNA sequences are available in the NCBI database under accession numbers KP677335, KP677336 and KP677337.

## Supplementary Materials

The following supporting information can be downloaded at: https://www.mdpi.com/article/10.3390/genes15020159/s1, Supplementary Table S1. List of primers used for the construction of KDEX and KDEXr; BTAXr; ACEX and ACEXr clones and for the PCR and qPCR detection of the BRUs. Supplementary Table S2: Repetitive DNA sequences obtained using KDEXr, BTAXr and ACEXr primers in different species used in the study. Supplementary Table S3. Distribution of BLASTN matches for KDEXr, BTAXr, and ACEXr sequences on X chromosomes of *B. taurus*, *C. hircus*, and *O. aries.*
