# Supplementary material for: X Chromosome-Specific Repeats in Non-Domestic Bovidae
Source: Genes (Basel). 2024 Jan 25;15(2):159. doi: 10.3390/genes15020159 (PMC10887555; doi:10.3390/genes15020159)
Supplement: Supplementary file 1 [file genes-15-00159-s001.zip › Supplementary Table S1.pdf]

**Supplementary Table S1. List of primers used for the construction of KDEXr, BTAXr and ACEXr clones, and for the PCR and qPCR detection of the BRUs.**

| Clones | Primers | |
|---|---|---|
| | Construction of clones | PCR/qPCR detection |
| KDEXr | CATGTGTTCCTGGTCCTGAACCC | GGCTTTGTGATGTCAAGGCTCACC |
| | TTCCTTTCTTCCCTCCTTCCTCC | CCTACTTTCAGGGCAAACTGGACC |
| BTAXr | GGGCAACAGACTGAGGATTCACC | GGGCAACAGACTGAGGATTCACC |
| | CTGTCCTGACTACTCTGGCTGTCC | CTGTCCTGACTACTCTGGCTGTCC |
| ACEXr | CCTCTGTCCATGCAATTATCCAGG | CAGCAGGGTACATGCACACTGAGG |
| | CTAGTGCTGTTTGTGGGAGTGGGC | AAGCGTCCAGCTTGGGTGTCGTGG |
