# Supplementary material for: X Chromosome-Specific Repeats in Non-Domestic Bovidae
Source: Genes (Basel). 2024 Jan 25;15(2):159. doi: 10.3390/genes15020159 (PMC10887555; doi:10.3390/genes15020159)
Supplement: Supplementary file 1 [file genes-15-00159-s001.zip › Supplementary Table S2.pdf]

KDEXr sequence

>*Redunca fulvorufula*
GGCTTTGTGATGTCAAGGCTCACCCAGGGCCCCCATTGGCTGGTGGAGAGCCTTGCTGAACTCATAAAGCATGAGGGTGTGTC
TCCCTGGGGACGGGGTATATCTAGGGGTGCTCAGGGGCTCTGGAGCAGACCACTGGCACGCCTCAAAATGACTAAAGTGACTG
GGAAGCCCCAAGGCTCCGGAGTAGCTAGGAAACACCTGCCTCCTCTTACCCCGGACAAAAGGATGAGACCTCCTCTCAACTGA
GGTCCAAGAAAAAGTCAAGGAGAGCTGAGCCCCACCCCAAAACTCTTACCCGTTGACCCCACCCCATGAGACCCCCTCCCCTA
TCTTGCCTBGCACAGAACCCTTCTCTGTCCACACCCTGCTCCTGAAGCTGTCATTCCCATCCACCTTCACCCTCTTCCCAAAC
CGGTGCCAATTCTCCACCCCTCCTCTGTTTTCTCCAGGTGGCCAGGGTAACCGTGAGAGTGAGAAATTCCCTAAGGGCAAAGC
TGACCAAAGGAACTCTTCAGAAACCACCACTCAAGGAAACCTTAAAAAATCAAGGGCTCCAAGCTCCGTAGCAGTGCTCCAAG
GTGGAATGAGAACTGATCAGACCGGACCCAGAAGAGGTTCCCCGAAGAGGCTGGAATCCCCTGGTCATTCAAGCAGGAAACAG
TGGGGCAAGCCAGGGACCTCAGGCATGGCTAAGAGACCTCAGACATCTTTCGTTTGTTCTTGCTTCGGAGTACTGCCCAACAG
TGCAGACGTTGACTATTTATACCGACTGCATACAGTCATAATGAAAATAAAATCCCCCTGACGTGAAATTATGTTTATCTCCT
AGTTCTGTGAGAGTGGGGGCAGAGAGGGCAGGCAGGCATAAATGTGTGAAGAGGGAGGGAGTGGGATGGGTCCTGTGTGGTTG
GAGATCGTACCAGAAGGTCCAGTTTGCCCTGAAAGTAGG

>*Aepyceros melampus*
GGCTTTGTGATGTCAAGGCTCACCCTTGGCCACCATTGGCTGGGGAAGAGACTTGCTGACCTCATAAAGCATGACGGTGTGTC
TCCCTGGGGAGGGGTATATATAGGGGTGCTCAGGGGCTCTGGTGCAGCCCACTGGGAGGCCTCAAAATGACTAAGGTGACTGG
GAAGCCACAAGGCTCCAGAGTAGTTATGAAACGCCTGCCTCGGGTTACCCTGCACAGAAGGATGAAGACCTCCTCTCAATTGA
GGTCCAAGAAAAATGTCAAGGTGAGCTGAACCCACCCAAACTCCTCTTGGCCATTGACCCCACCCCATAAGACCCCCTCCCCC
GTCCTTGCCTGGCACATAACCCTTCTCTGCCCACACTCCTTCTCCTGAAGCTGTCATTCCCATCCACCTTCACCCTCTTCCCC
AAACCAATGTCATTCTCTACCCCTCTCTTGTTTTCTCCAGGTGGCCAGGGTAACTGTGAGAGTGAATAATCACCTTCAGGCAA
AGCTGACCAAGAAAACTTTTCAGAAACCACCCACCACAAGGAAACTTTAAAAAGTTAGAGGCTCAAAGCTCTGTAGCCAGTGC
TCCAAGGTGAATGAAGAGTTGAATCAGAACGGACCAGAGGAGGTCCCAGAGACTGTGGAGACCCCTGTCATTCCAGCAGGACC
AGTGGGCAGCCAGTGACCTCAGAGCATGGCTAGAGACCTCAGAAATCTTCGGGGGTCTTGCCACTGAGTACTGGCCAACAGTA
CAGACATTGACTAGTATAGCAACTGCATACATTCATAATGAGAATAAAATCAACCTGATGTGAAATTATGTTTGTCTCCGAGT
TCTGTGAGAGTGGGGGGCGGAAAGGGCAGGGCAGGCATAAATGTGTGAAGAGGAAGGGAGATGGGTCCTGTGGGGTTGGAGAT
GGGACCAGAAGGTCCAGTTTGCCCTGAAAGTAGG

>*Cervus elaphus*
GGCTTTGTGATGTCAAGGCTCACCCAGGGCCACCATTGGCTGGGGAAGAGAATTGCTGACCTCATAAAGCATGAGGGTGAGTC
TCCATGGGGACAGGTATATATAGGGGTTCTCACGGGCTCTGAGCAGACCACTGTGAGTCCTCCAAATGAACGGGGTGACTGGG
AAGCCACAAGGCTCCAGAGTAGTTATGGAACACCCACCTACTGTTACCCAGGAGAAAATGAATACCTTTTATCAATTGAGGTC
CAAAGAAATGTCAAGGTGAACTGAACCCAGCCAAACTCCTCTTGCACATTGACCCCACCCCAGAAGACACCCTCCCCTGTCCT
TGCCTGGCACATAACCCTTCTCTGCCCACACCCCTTCTCCTGAAGCCATCGTTCCCACTTGTCTTCACACTCTTCCCTAAACC
AGTGCAATTCTTGCTCCCTGTCTTGTTTTCTCCAGGTGGCCAGGGAAAACGTGAGGGTGAATAATCCCATTCCGGCAAAGCTG
CTCAAGACAACTTCTCTGAAAACTCCAACAAAAAAGCTTAAGATAATCAGATGCTCAAAGCTTTGTAGCCAGAGTTCCAAGGC
AATTGAACAGCTGAAACGGAAGGAAATGGAGGAGGTCCCAGAGACCATGGAGACCCCTGCCATTCCAGCTAATCAGAATGAAT
CAGAGGAGGTACCAGAGACCATGGAGATCCCAGCCATTCCAGCCTATCATAATGAACCAGAGGAGGTCCCAGAGACCATGGAG
GCCCCTGCATTTCCAGCTAACCAGAATGAACCAGAGGAGGGCCCCGAGACTGTGCAGACCCCTGCCATTCCAGCGAATCAGAA
CGAACGAGAGGAGGTCCCAGAGACCATGGAGACCCCTGTCATTCCAGCTAATCGGAATGAACCAGAGGAATTCCCAGAGATCT
TGGAAACCCCTGCCATTCCACCTTTACCAGTGGGCAGCCAGTGAATTCGGGGCATGGTTAGACACCTTAGAAATGCTTGCGGG
TCTGGCCGCTGAGTACTGGCCAAAAGTACAGATGCTGAATAGTATAGCCACTGCATACACTAATAGGGAAATAAACTAAAATC
AAGCTGATGTGAACTTATGTTTGTCTCTGAGTTCTCTGATAGTGGGAGGCAGAGAGGGCAGGGGAGGGATAAGTGTGTGAGGA
GGGAAGCAGATGGCTTCTGTGGAGTTGGACATCGAACCAGAAGGTCCAGTTTGCCCTGAAAGTAGG

>*Antilocapra americana*
GGCTTTGTGATGTCAAGGCTCACCCAAGGCCACCATTGGCTGGGGAAGAGACTTGCTGACCTCATAAAGCATAAGGGTGTGTC
TCCCTGGGGAAGGGTATATATAGGGGTGCTCAGGGGCTCTGGAGCAGACCACTGGGAGGCCTCAAAATGACTAAGGTGACTGG
GAAGCCACAAGGCTCCAGAGTAGTTAGGAAACATCTGCCTCCTCTTACCCAGGACAAAAGGAGGAAGATCTCTCAACTGAGGT
CCAAGAAAAATGTCAAGGTGAGCTGAACCCACCCAAACTCCTCTTGCCCATTGATCCTACCCCATGAAGACCCCCTCCCCTGT
CCTTGCCTGGCACATAACCCTTCTCTGCCCACACCCCTTCTGAAGCTGTCGTTGCCGTCCACCTTCACCCTCTTCCTCAAACC
AATGCCATTCTCTACCCCTCTCTTGTTTTCTCCAGGTGGCCAGGGTAACTGTGAGAGGGAATAATCACCTTCAGGCAACACTG
ACCAAGAAAATGCCTCAGAAACCGCCCACCACAAGGAAACTTAAAAAAATCAGAGGCTCAAAGCTCTGTATCCTGTGCTCCAA
GGTGAATGAAGAGCTGAATCAGAACGCACCAGAGGAGGCCCCAGAGAGTGTGGAGACCCCTGGCATTCCAGCAGGAGCGGTGG
GCACCCAGTGACCTCAGAGCATGGCTAGAGACCTCAGAAATCTTCGGGGGTCTTGCCGCTGAGTACTGGCCAACAGTACAGAC
GTTGACTATTATACCAACTGCATACATTCATAATGAAAATAAAATCAACTTGATGTGAAATTATGTTTGTCTCCGAGTTCTGT
GAGAGTGGGGGGCAGAGAGGGCAGGGAAGGCATAAATGTGTGAAGAGGGAGGGAGATGGGTCCTGTGGGGTTGGAGAGGGGAC
CAGAAGGTCCAGTTTGCCCTGAAAGTAGG

BTAXr sequence

>*Nanger dama*

GGGCAACAGACTGAGGATTCACCAAGTCTTGGGGTCTCTAATCCTCATTGGAGTAGAAAAAACATCCAACCTGTTCTTAATTA
CCTTTTTCCATGTTACAGGCTTGCTCTTAAGTTCCTTAAGGCCCTCAAGAACCCTCAATAGGAAGAGGGTGGTGTAAAGATGA
AGTGACTAGAGGACTCTGAAGTGCTAAAATGTGATGAAAAGTGTTTGGCAGAGGTTCATACAGGGATGGAGGAGGCAGAAGTG
TTGAAGGGGGACATATAAGTCAAAGAATAAAGGGCACTGAAGGAAAGTCTGAGGGTCACGAGAAGAAAAACCAAGCCCCTGAA
TCACCGCTCCCCAGAATCAGACACACAGAGGTGTCAGGGGTGGGAAAGTGGTATCACGGCCTCCCTCAGCACCCATGAACCCA
TGTTGATGGGCACAGTGGTACATTGTGATGTCTGAGACCCCAAAGCCACCATTGGCTGGGGGAGTGCCTAGCTGACCAATGAG
GTTGAAGGGTGTGGCTCCTCATTGTCCTGGGAAGGTATATATAGGGAGCTCAGAGGCAGAGATTCTGGGTTAGGCCACTTCAC
GGTGTCATCATGGCTAAGGTAACCAGGAAGCCACGGCAGCCAAGAAGAGTTGCAATGCGCTTTGCTTCAAGGATGAAAGGAAG
AAAGAGGACCCTTTGTCGACGGAGATACAGAGGCAGTGTGAAGGTAAGATGATTCTCTCTACAGTCCTCACGTTCTCCATTGA
CTTTGCTGCCCAAGACCTCTACACTGCCCTTGCCTGGCACAAAAGTCCTCATCAGGCCACATCCCTTTTCATGAAATCTCCCT
TCCAACTCAGTTGTTATACTCTTTGCTCAAAACTAATACCCTTCTCTTGTCTTTCAAGGCACGAAATATGACCATGAGGGTCA
GAAGACCTCGAAAAGGAACCTTGAGAAAGAAAATCCGATCATATTCCACTCCGTCAAAGAAGGTGAAGAAAACAAGAAAACCA
AATGGTTTTTTCTGTTCCTGTGCACATAAGAAACTGAATCAAAGCCTAAAAAGGTACCCAAATACGAGGCAGAGTCAAAGAAG
GAGGCAGAATCAAAAGAGAAGATAAGCTCTGCCACCCCAGAATGAGAGACCCTGGAGATGTACAACCTGGGCAGCCAGTAACT
GAATAGAAAAAGTTAGACAGTTTAGAATTGTGACTCCAGTGGTCTGCTTTCTTAGAAATGGCCAACCAGGAAAAGACTGGCTC
TCATACTAACATAGTAAACTGATGGCTGCGATTAAAATAAACATCAAATGGTGAAAAGATGATATTTGTGTGTGAGTGTGAAT
TTTCTGATGGGAAGGGAGGGAAGGAAGAAAAGAGATGGGCACCTGAGTGGGTTGGGATGGGTCCTGAGAGATTGGGGGACAGA
CCCTGAGGTCCCCAGTTAGCCAGAGTAGAGGTCTGGACTGAGTCAGGAGTCTGCACTGAAGATGGATAACCCTGCTTAACACT
TGATCTCAGCGGCAAGGAGTAGTGGTGTGGTGTCACTGCCATTGTTTGGGGTGTGGAATGACATTAGGGTCTATATTGCCAGA
ACCCAGATGGGAAGAGGTTTTCACACAGGGATGGTGAATGTCATACTTCCCCCAAGGGAGGCAGGCAGAAATCACATCCTGGC
CACTTACATCATAAAGGGCTCTGTTGCATCCCTCCCCTATATTGTCAGCTGAAGGTGCTCAGGAGGCACAAAGTGCTGATACA
ACTAAAACCCACAACTAGCACTCCCAATGATTACAATGATGTTTGGAGGGAACAGACCAAATATCAATTAGGCCACTGTCATC
CTAAGAAATTTGTGGGAGCAGTGTGTTCCCAAGGGCAGGGCAGTGGAGGTGGCTCAATCCCAGTGCAGATGACACACAGGATC
ACCACACAGAGGTAACCTTTTTGGGTGGGTGGGCACCATGAAGTTTGTGGGAACTTTGTTCCCCGACCAGAGATTGAACCGGT
TCCCCTTCTAGTGAAAGCAGGGAGTCCTAATCCCTGGACAGCCAGAGTAGTCAGGACAG

>*Aepyceros melampus*

GGGCAACAGACTGAGGATTCACCAAGTCTTAAAAACATACAAACTGTTCTTAATTACCTTTTTCCATGTTAAATGCTTCCTAT
TAAGTTCCTTAAGACCCTCAAGAACCCCCAATAGGAAAAGGGTGCTCTAGAGATGAAGTGACTAGAGGACTCTGAAGTGCTGA
AATGGGATGAAAAGTGTTTGGCAGAGGTTCATACAGGGATGGAGGAGGCAGGACTGTTGAATAGGGACATAAAAGTCAAAAAA
TAAAGGGCACTGAAGGAAAGTCGGAGGGTCATGAGAAGAAACACCATGCCCCTGAATCACCTCTCCCCAGAATCAGACACACA
GAGGGGTCAGGAATGGGAAAGTTATATTATGGCCTCCCTCAGCATCCACTAGCCCATGTTGATGGGAAAAGTGGTACATTGTG
ATGTCTAAGACCCCAAAGCCACCATTGGCCAGGGGAGAGGCTAGCTGACCAATCAGATTGAAGGGTGTGGCTCCTCATTGTCC
TAAGAAGGTATATATAAGGAAGATAGAGGCAGAGATTCTGAGTTAGGCCACTTCATGGTATCATCATGAGTAAGGTAACCAGG
AAGCCATGGCAGCCAAGAAGAGTTGCAATGCGGTTTGCTTCAAGGATAAAAGGAAGAAAGAAGACCTTTTGTCAATGGAGATA
CAGAGGCAGTGTGAAGGTAAGATGATTTCATCTATGCTCCCCATGTTCTCCATTGACTTTGCTGCCCAAGACCTCTACACTGC
CCTTGTCTGGCACAAAAGTCCTCATCAGGCCACATCCCTTTCATGAAATCTTCCTTCCAACTCAGATGGTATACTCTTTCCTG
AAAACTAATACACTTCTCTTGTCTTTTCAGGCACGAAATATGACCATGAGGGTCAGAAGACCTTTACAAGGAAACTTGAGAAA
GAAAATCCGATCATATGCCACTCAATTGAAGAAGGTGAAGAAAAAAAACAAAACTGTTTTTTCTATTGCTGTGCACGTAAGAA
ACTGAATCAAAGCAGAAAAAGGTACCAAAAATAGGAGGCAGAATCAAAGAAGAAGGCAGTATCAAAAGAGAAGATAAGCTCAG
CTGTCCCAGAATAAGAGACCCTGGAGAAGTACAACCTGGTCAGCAAACAACTAAATAGAAAGTATCAGACAGTTTAGAATTGT
GTCTCCAGAGGTCTGCTTTCTTAGAAATGGCCAACCAGGAAAAGACTTACTCTCACACTAACATAGTAAACTGATGGCTGCAA
TTAAAATAAACAAATGGTGAAAAGATTACATTTGTGTGTGTGTGTGAATTTTCTGATGGGAAGGGAGGGAAGGGAGGGAAGGA
AGAGAAGAGCTCGGCACTTGAGTGGGGAAGGATGTGGGGTCCCAGGAGATTGGAGAACAGACCCTCAGGTCCCCAGTTAGCCA
GAGAGTAGAGATTTGGACTGGGTCAGGAATCTGCACTGAAGATGGATAACCCTGCTTAACAATTGACCTCAGTGGTAAGGAGT
AGTGGTGTGGTGTTACTGCCTTTGTTTTGGGTGTGGAATGATATTAGGGTCTAGATTGCCAGAACCTAGATGGGAAGAGGGTT
TCACATGGGGATGGTGAATGTCATACTTCCCCTGAGAGGCAGGCAGTAATCACATCCTGGCTACTTACGTCATAATGGGCTTA
GTTGCATCACTCAACTATGCTGTCAGCTAAAGGTGCTCAGAAGGCACAAAATGCTGATACAACTAAAATCCACAACTAGCACT
CCCAAAGATTAAAATAATGTTTGGAGTGAACAGACCAAATATCAATTAGGCCACTGTTATCCTAAGAAATTGGTTGGAGCAGT
GTGTTCCCAAGGGCAGGGCAGTGGAGGTGGCCCAATCCCGGTGCAGATGACATACAGGATCACCCCACACAGATAATTTTTTT
GGGTGGGGGGCACCATGCAGTTTGTGGGATCTTAGTTCCCTGACCAGGGACTAAACTGGTTCCCCTTCTAGTGAAAGCACAGA
GTCTGAACCACTGGACAGCCAGAGTAGTCAGGACAG

>*Ammelaphus imberbis*

GGGCAACAGACTGAGGATTCACCAAGTCTTGGGGTCTCTAAAGCTCATTGGAGTAGAAAAAACATCCAACCTGTTCCTAATTA
CTGTTTTCCGTGTTAAGTGCTTGTTCTTAAGTTCCTTAAGCCCCTAAAGAACCCCCAATAGGAAGAGGGTGGTTGAAAGATGA
AGTGACTGGAGGACTCTGAGGTGCTGAAATGGGGTGAAAAGTATGTGGCAGAGGTCCATACAGGGACGGAGGAGGCAGAAGTG
TTTAAGGGGGACATATAAGTCAAAGGATAAAGGGCACTGAAGGAAAGTCTGAGGGTCATGAGAAGAAACACCAAGCCCGTGAA
TCACCACTCCACAGAATCAGACACATAGAGGTGTCAGGATTGGGAAAGTTGTATCATGGCCTACCTCAGCACCCACTAACCCA
TGTTGATGGGCACAGTGGTACATTGTGATGTCTAAGACCCCAAAGCCACCATTGTCTGGGGGAGTGCCTAGCTGACCAATCAG
ATTGAAGGGTGTGGCTCCTCATTGTCCTGAGAAGGTATATATAGGGAGGTCAGAGGCAGAGAGTCTGGGTTAGGCCACTTCAT
GCTATCATCAGGCTAAGGTAACCAGGAAGCCACGGCAGCCAAGAAGAGTTGCAATGAGGTTTGCGTCAAGGATGAAAGGACAA

AAGAAGACCCTTTGTCAACAGAGATATAGAGGCAGTGTGAAGGTAAGATGATCCATCTATGCTCTCCACGTTCTCCATTGACT
TTGCTGCCTAAGTCCTCTATACTGCCCTTGCCTGCCACAAAAGCCCTCACCAGCCCACATCCCTTTTCATGAAATCTCCCTTC
CAATTCAGTTGTTATACTCTTTCTTCAAAACTAATAACCTTCTCTTGTCTTTCAAGGCACGAAATATGACCATGAGGGTCAGA
AGACCTCTAAAAGGAACCTTGAGAAAGAAAATCCAATCATGCCACTCAGTCCAAGAAGGTGAAGAAAACAAGAAAACCAAACT
GTTTTTTCTGTTCCTGTACACGTAAGAAACTGAATCAAAGCCAAAAAGGGTACCAAAATATGAGGTGGAGTCAAAGAAGGAGG
CAGAATCAAAAAGAGAAGATAAGCTCAGCCACTTCCAGAATGAGAGACCCTGGAGAAGTACAACCTGGGCAGCCAGTAACTGA
ATAGAAAGTCAGACACATTAGAATTGTGTCTCCAGAGGTCTGCTTTCTTAGAAATGGCCAACCAGGAAAAGACTGACTCTCAC
ACTAACATAGTAAACTGATGGCTGCAATTAAAATGAGCATCAAATGGTGAAAAGATGATATTTATGTCTGTGTGAATTTTCTG
ATGGGAAGGGAGGAAAGGAAGAAAAGAGGTGGGCACCTGAGTGGGGAGGGATGTGGCATTCCAAGAGATTAGGGAACAGACCC
TCAGGTCCCCAGTTAGCCAGAGAGTAGAGGTTTGTACATGGTCAGAAGTCTGGACTGAAGATGGATAACACTGCTTAACAATT
GATCTCAGCGGCAAGGAGTAGTGGTGTGGTGTTACTGCCATTGTTTGGGGTGTGGAATGACATTAGGGTCTAGATTGCCAGAA
CCCAGACGGGAAGAGGGCTTCACATGGGGATGGTGAATGTCATACTTCCCCTGAAAAGGCAGGCAGAAATCACATCCTGGCCA
CTTACGTCATAGTGGTCTTTGTTGCATCACTCACCTATGCTGTCAGCTAAACGTGCTCAGGAGGCAAAAAATGCTGATACAAC
TAAAACCCACAGTTAGCACTCCCAAAGATTAAAATAATGTTTGGAAGTAACAGACCAAACATCATTTAGCCCACTGTTATCCT
AAGAAATTGGTGGGAGCAGTGTGTTCCCAAGAGCAGGGCAGTGGAGGTGGCCCAATACCGGTGCAGATGACAAACAGGATCAC
CACACACTGATAATTTTTTGGGGGGTGGGGGTCACCATGCAGTTTGAGGGATCTAAGTTCCCTGACCAGGGATTGAACCGGCT
CCCCTTCTAGTGAAAGCACGGAGTCCTAACCACTGGACAGCCAGAGTAGTCAGGACAG

>*Hippotragus equinus*
GGGCAACAGACTGAGGATTCACCAAGTCTTGGGGTCTCTAAAGCTCATTGGAGTAGAAAAAACATCCAACCTGTTCTTAGTTA
CCTTTTTCCATGTTAAGTGCTTGCTCTTAAGTTCCTTAAGCCCCTCAAGAACCCTCAATAGGAAGAGGGTGGTCTAAAGATGA
AGTGACTAGAGGACTCTGAAGTGCTGAAATGGGATGAAAAGTGTTTGGCAGAGGTTCATACAGGGATGGAGGAGGCAGGAGTG
TTGAAGGGGGACATATAAGTCAAAGAATAAAGGGCACTGAAGGAAAGTCTGAGGGTCATGAGAAGAAACACCATGCCCCTGAA
TCACCACTCCCCAGAATCAGACACACAGAGGTGTCAGGGGTGGGGAAGTTGTATCATGGCCTCCCTCAGCACCCACTAAGCCA
TGTTGATGGGCACAGTGGTACATTGTGATGTCTAAGACCCCAAAGCCACCATGGCTGGGGGAGTGCCTAGCTGACCAATCAGA
TTGAAGGGTGTGACTCCTCATTGTCCTGGGAAGGTATATATAGGGAGGTCAGAGGCAGAGATTCTGGGTTAGGCCACTTCATG
GTGTCATCATGGCTAAGGTAACCAGGAAGCCACAGCAGCCAAGAAGAGTTGCAATGCGGTTTGCTTCAAGGATGAAAGGAGGA
AAGAAGATCCTTTGTCAACGGAGATACAGAGGCAGTGTGAAGGTAAGATGATTCCATCTACACTCCTCATGTTCTCCATTGAC
TTTGCTGCCCAAGACCTCTACACTGCCCTTGCTTGGCACAAAAGTCCTCATCAGGCCACATCCCTTTTCATGAAATCTCCCTT
CCAACTCAGTTGTTATACTCTCTCCTCGAAACTAATACACTTCTCTTGTCCTTCAAGGCACGAAATATGACCATGAGTGTCAA
AAGACCTCTAAAAGGAACCTTGAGAAAGAAAATCCGATCATACGCCACTCCGTCGAAGAAGGTGAAGAAAACAAGAAAACCAA
ACGGTTTTTTCTGGTCCTGTGCACGTAAGAAACTGAATCAAAGCCAAAAAGGGTACCAAAATACGAGGCAGAGTCAAAGAAGG
AGGCAGAATCAAAAGAGAAGATAAGCTCTGCCACCCCAGAATGAGAGACCCTGGAGAAGTACAACCTGGGCAGCCAGTAACTG
AATAGAAAAGGTCAGACAGTTTAGAATTGTGTCTCCAGAGGTCCGCTTTCTTAGAAATGGCCAACCGGGAAAAGACTGACTCT
CACACTAACATAGTAAACTGATGGCTGCGATTAAAATAAACATCAAATGGTGAAAAGATGATATTTGTGTGTGTGTGAATTTT
CTGATGGGAAGGGAGGGAAGGAAGAAAAGAGGTGGGCACCTGAGTGGGGAGGGGTGCGGGGTCCCGAGAGATTGGGGAACAGA
CCCTGAGTTCCCCAGTTAGCCAGAGAGTAGAGGCCTGGACTGGGTCAGGAGTCTGCACTGAAGATGGATGACCCTGCTTAACG
ATTGATCTCAGCGGCAAGGAGTAGTGGTGTGGTGTTACTGCCATTGTTTGGGGTGTGGAATGACATTAGGGTCTAGATTGCCA
GAACCCAGATGGGAAGAGGGTTTCACGTGGCGATGGTGAATGTCATTCTTCCCCTGAGAGAGGCAGGCAGAAATCACATCCTG
GCCACTTAATGTCATAATGGGCTTTGTTGCATCACTCACCTATGCTGTCAGTTAAAGGTGCTCAGGAGGCACAAAATGCTGAT
ACAACTAAAACCCACAATTAGCACTCCCAAAGATTAAAATAATGTTTGGAGGGAACAGACCAAATATCAATTAGGCCACTGTT
ATCCTAAGAAATTGGTGGGAGCAGTGTGTTCCCAAGGGCAGGGCAGTGGGTGTGGCCCAATCCCAGTGCAGATGACAAACAGG
ATCACCACACACAGATAATTTTTTTGGGTGGTGGGCACCGTGCAGTTTGTGGGATCTTAGTTCCCTGACCAGGGATTGAACCG
GTTCCCCTTCTAGTTAAAGCATGGAGTCCTAACCCCTGGACAGCCAGAGTAGTCAGGACAG

>*Cervus elaphus*
GGGCAACAGACTGAGGATTCACCAAGTCTTGGGTTCTCTTAAAGCTCAATGAAGTAGAAAAAACATCCAACCTGTTCTTAATT
ACCTTTTTCCATGTTAAGTGCTTACTCTTAAGTTCCTTAAGCCCCTCAAGAGCTCCCAACAGGAAGAGGGTAGTCTAGAGTTG
AAGTGACTAGAAGACTCTGAAGTGCTGAAATGGGATGAAAGGTGTTTGGCAGAGGTTCATATAGCGATGGAGGAGGCTCACAG
GGAGATGGAAGAGGCAGGAGTGTTGAAGGCAGACATATAAGTCAAATAATAAAGGGAACTGGAGGAAAGTCTGAAGGTCATGA
GAAGAAGCACCATGTCCCTGAATCACCACTCCCCAGAATCAGACACACAGAGGTGTCAGGATTGGGAAAGTTATATCATGGCC
TCCTTTAGCATCCATTAGCCCATGTTGATGGGCAAAGTGGTACCTTGTGATGTCTAAGACCCCAAAGCCACCATTGGCTGGGG
GAGTGCCTAGCTGACCAATAAGATTGAAGGGTGTGGCTCCTCATTGTCCTGAGAGAGTATATATAGGGAGGTCAGAGGCAGAG
ATTCTGGGTTAGGCCACTTCATGGTATCATCATGACTAAAGTAACCAGGAAGCCACGGCAGTCAAGAAGAGTTGCAATGTGTT
TTGCTTCAAGGATGAAAGGAATAAAGAAGACCCTTTGTCAACGGAGATACAGAGGCAGTATGAAGGTAAGATGACTCCATCTA
TGCTCCCCATGTTCTCCATTGACTTTGCTGCCCAAGACCTCTACACTGCCTTTGCCTGGCAAAAAGCCCTCAGCAGCCCACAT
CCCTTTTCATGAAATCTCCTTCCAACTCAGTTTTTCTACTTTTTCCTCAAACTAATACCTTCTCTTGTCTTTCCAGGCACAAC
ATATGACCATGAGAGTCAGAAGACCTCTAGAAGGAAACTTGAGAAAGAAATTCTTATCATATGCCACTCAGTCGAGGAAGGTG
AGGAAAACAAGAAATCCAAACTGTTTTTTCTGTTCCTGTGCACGTAAAAAACTGAATCAAAGCCGAAAAAGGTACCAAAATAT
GAGGCAGAGTCAAAGAAGGAGGCAGAATCAAAAGAAAAGATAAGCTCAGCTGCCCCAGAATAAGAGACCCTGGAGAAGTACAA
CCTGGGCAGCCAGTAAGTGAATAGAAAAGGTCAGACAGTTTAGAGGTGTGTCTCCAGAGGTCTGCTTTCTTAGAAATGGCCAA
CCAGGAAAAGACTGACTCTCACAGTAACATAGTAAACTGATGGCTGAATTAAAATAAACATCAAATGGTGAAAAGATGATATT
TGTGTGTGTGTGAATTTTCTGATGGGAAGGGAGGGAAGGAAGAGAAGAGGTGGGCACCTGAGTGGGGAGGGATGTGGGGTCCG
AGGAGATTGGGGAACAGACCCTCAGGTCCCCAGTTAGCCAGAGAGTAGATTTCTGGACTGGGTTGGGAGTCTGCACTGAAGAT
GGATAACCCTGCTTAACAATTGATCTCAGTGGCAAGGAGTAGTGGTGTGGTATTACTGCCTTTGTTTGGGGTGTGCAATGACA
TTAGAGTCTGGATTGCGGAACCCAGATGGGAAGAGGGTTTCACATGGGGATGGTGAACGCCATACTCCCCCTGAGAAAGGCGG
GCAGTAATCACCTCCTGTCCACCTATGTCATAATGGGGTTTCTTGGATCACTTGCTTTGCTGTCAGCTAAAGGTGCTCAGGAG
GCACAAAATGCTGATACACCTAAAACCCACAACTAGCACTCCCAAGGATTAAAATAATGCTTGGAGGAAACAGACCAAATAAC

AATTAGGCCACTGTTATGCTAAGAAAGTAGTGGGAGCAGTGTGTTCCCAAGGGCAGGGCAGTGGAGGTGGCCCAATCCTGGTG
CAGATGACAAACAGGATCACCACACACAGATAATTTTTTTGGGTGGGGGAACCATGTAGTTTGTGGGATCTTAATTCCCTGAC
CAGGGATTGAACCAATTCCCCTTCTGTTGAAAGCATGGTGTCTTAACCACTGGACAGCCAGAGTAGTCAGGACAG

>*Giraffa camelopardalis*
GGGCAACAGACTGAGGATTCACCACGTCCTGGGGTCTCTAAAGCTCACTGGACTAGAAAAAACACCCAACTTGTTCTTAATTA
CGTTTTTCCACCTTAAGTGCTTGCTCTTAAGTTCCTTAAGCCCCTCGAGAACACCCAACGGGAAGAGGGTGGTCTAGAGATGA
AGTGACTAGAGGATTCTGAAATGCTCCAATGGGATCAAAAGTGTTTGGCAGAGATTCAAAGAGGGATGGAGGAGGCAGGAGTG
TTGACGTAGCACATATAAGTCAAAGAATAAAGGATACTGAAGGAAAGCCTGAAGGTCATGGAAGAAACACCATGTCCCTGAAT
CACCACTCCCCAGAATCAGCTACACAGAGGTTGTCAGGAGTGAGAAAGTTGTATCATGGCCTCCCTCAGCATCCGCTAGCCCG
TGCTGATGGGCACAATGGTACAGTGTGACGTCTACGACCCCAAAGCCACCATTGGCTGGGGGAGTGCCCCTAGCTGACCAATC
AAATTGAAGGGTGTGGCTCCTCATTGTCCTGAGAGGGTATAAATAGGGAGGTCAGAGGCAAAGAGTCTGCGTTAGGCCACTTG
TTGGCATAATCATGACTAAGGGAACCAGGAAGCGACGGCAGTCAAGAAGAATCGCAATGCAGTCTGCTTCAAGGATGGAAGGA
AGAAAGAAGGCCCCTTGTCATCCGATGTGCAGAGGCAGTGTGAAGGTAAGATGACTCCAACTAAGCTCCCCATGTACTCCATT
GACTTTGTTGCCCAAGGCCTCTATGCCGCCCTTGCCTGGCACAAAAGCCCTCATCAGCCCACATCCCTTTTTCATGAAATCTC
TCTTCCAACTCAGTTTTTATACTCTTTCCTCAAGACTAATACCCTTCTTTTGTCTTTCCAGCCACGAAAGGTGACCAAGAGGG
TCAGAAGATCTCTACAAGGCACCTTGAGAAAAAAATCCAATCAGATGCCACTCAGTCGAAGATGGTGAAGAAAACAAGAGATT
CAAACTCCTGTCTCTGTTCCTGTGCACGTAAGAAATTGAATCAAATTGGAAAAAGATACCAAAATAGGGTCAGAGTCAAAGAA
GGAGGCAGAATCAAGAGAAGATAAGTTCAGCCACCCCAGAATAAGAGACCCTGGAGAAGTGCAACCTGGGCAGCCAGTAACGG
AATAGAAAAGGCCAGACAGTTTAGAACTGTGTCTCCAGAGGTCTGCTTTCTTAGAAATGGTCAACCAGGAAAAGACTGACTCT
CACTCTAGCATAGTGAACTGATGGCTGCGATTAAAATAAACATCAAATGGTGAAAAGATGACATTTGTGTGTGTGCGAATTTT
CTGATGGGAAGGGAGGGAAGGAAGAGAAGAGGCGGGCACCTGAAAAGGGAGGCACATAGGGTCCCGGGAGATTGGGGAACAGA
CCCTCAGGTCCCCAGTTAGCCAGAGAGTGGAGGGCTGGACTGGGTCAGGAGTCTGCACTGAAGATGGATAGCCCTGCTTAACA
ATTGAACTCAGCGGCAAGGAGTAGTGGTGTGGTATTACTGCCTTTGTTTGGGGTGTGGAATGACATTAGGGTCTAGATTGCCA
GAACCCAGATGGGAAGAGGGTATCTCATGGGGATGGCGAATGTCATACTTCCCCTGAGAATGGCAGGCAGAAATCACCTCCTG
GCCACCTACCTCATAATGGGGTTTGTTGCATCACTCGCCTATGCTGTCAGCTAAAGGTGCTCAGGAGGCACAAAATGATGATA
CAACTAAAACCCTTAACTATCACTCCCAAGGATTAAAATAATGTTTGGAGGCAACAGATCAAATATCGCTCAGGCCACTGTTA
TGCTAAGAAATTGGCAGGAGCAATGTGTTCCCAAGGGCAGGGCAGTGGAGGTGGCCCAATCCCAGTTGAGATGACAAACAGGA
TCACCACACACAGAAAATTTTTGGGGGCGGGGGCCACCATGCAGGTTGTGGGATCTTAGTTCCCTGACCAGGGGTTGAACCGG
TTCCCCTTCTAGTGAAAGCATGGAGTCCAAACCACTGGACAGCCAGAGTAGTCAGGACAG

>*Antilocapra americana*
GGGCAACAGACTGAGGATTCACCAAGCCTTGGGGTCTCTAAAGCTCATTGGAGTAGAAAACACACCCAACCTGTTCTTACCTA
CTTTTTTCCACCTTAGGTGCTTGCTCCTAAGTTCCTTAAGCCCCTCAAGAACCCCCACCAGGAAGAAGGTGGTCTAGAGATGA
AGTGACTCGAGGAATCGGAAGGGCTGAAAAGGGATGCAAAGTGTTTGGCAGAGGTTCCTACAGGGATGGAGGAGGCAGGTCTG
TTGAAGGGGGACATCTAAGTCCAAGAATAAAGGGCACTGAAGGAAAGTGTGAAGGTCATGAGAAGAAACCCCATGCCTCTGAA
TCACCACTCCCCAGAATCAGCTATGCAGCACTTGTCAGCAGTGGGAAAGTTGTATCATGGCCTCCCTCAGCATCCACTAGGCC
ATGTTCATGGGCCCAATGGTACATTGTGATGTCTAAGGCCCCAAAGCCACCATTGGCTGGGGGGTGTGCCTAGCTGACCAATC
ACATTGAAGGGCGTGGCTCCTCATTGTCCTGAAAGGGTATATATAGGGAGATCAGAGGCAGAGATTCTGAGTTAGGCCACTTC
TTGGCATCAACATGACTAAGGTAACCAGGAAGCCACAGCAGCCAAGAAGAGTTTCAAAGAGGCTTGCTTCAGCAGTGAAAGGA
AGAAAGAAGCCCCTTTGCCTTCGGACCCTTGGTCATCAGAGGAACAGAGGCAGGGTGAATGTAAGATGACTCCAGCTAAGCTC
CCCATGCACCCCATTGGCTTTGGTCCCCAAAGCCTCTGTGCTGCCCTTGCCTTGTACAAAAACTCTCCTCAGGCCACATCCCT
TTTTCATGAAATCTCCCTTCAAACTCAGTTTTTACACCCTTTCCTCAAAACGAATACCCTTTCCTTGTCTTTCCAGGCACGGA
AGATGACCATGAGTGTCAGAAGACCTCTACACGGAACCTTGAGAAAAAAAATCCTGTCATACGCCACTCGGTCAGAGAAGGTG
AAGAAAACCGAAAAACCAACCTGTTTTTCCCGATCCTGCACACGTAAGAAATTGAAACAAAGCAGGAAAAGGTACCAACGTAT
GAGGCAGAGTCGAAGAAGGAGGCAGAATCGAAAGAGAAGACAAGCTCCGCCGCCCCAGGAGAAAGAGACCCTGGAGAAATACA
ACGTGAGCAGCCAGTAACTGAGTAGAAAAGGCCAGTTTAGAATTGTGTCTCCAGAGGTCTGCTTGCTTTGAAATGGCCAACCA
GGAAAAGACTGACACTCACACTGACATACTCAACTGATGGCTGCAATGAAAATAAACACCAAAAGGTGAAAAGATGATATTTC
TGTGTGTGGTGTGAATTTCCTGATGGGAAGGGAGGGAAGGAAGGAAGAGAAGAGGTGGGCACCTGAAAAGGGAGGGATATAGG
GTCCCGAGAAGTTGGGGAACAGACCTTCAGGTCCCCAGTTAGCCAGAGAGTCGAGGTCCGGACTGGGTCAGGAGTCTGCCCTG
AAGATGGACAACCCTGTCTAACAATTGATCTCAGCAGCAAGGAGTAGTGGTGTGGTGTTACTGCCTTTGTTTGGGGTGTGGAA
TGACATTAGGGTCTCGATTGCCAGACCCAGGTGGGGGGAGGGTTTCACATGGGGATGGTGAATGTCATACTTGCCCTGGGAGA
GGCAGGCAGAAATCACCCCCTGGTGCTTTCTGAGTGCTCAGGAGGCACAGACTGCTGATACAACTAAAACCCACAACTATCCC
TCCCAAGGATTTAAATAATGTTTGGAGGTAACAGACCAAATATCAGTTAGGCCACTGTTATGCTAAGAAAGTGGTGGGAGCAG
TGTGTTCCCAAGGGCAGGGTAGTGGAGGTGGCCCAATCCCGGTGCGGAGGACAAAGAGGATCACCACACACAGGTAATTTGGG
GTGAGTAGGGGGCACCACACAGGTTCTGGGATCTTATTTCCCTGACCAGGATTTGAACCAGCTCCCTTGATAGTGAGAGCACG
GAGTCCTAACCACTGGACAGCCAGAGTAGTCAGGACAG

ACEXr sequence
>*Madoqua kirkii*
CAGCAGGGTACATGCACACTGAGGCCCAGGATAGAACTTCCAGACCTCAAGGGTCATTAGGTTCTGTGGTGGGGGCACTTGGG
GCTGAGTCCATAATGAGGCATTAAATGACTTGGCCAGCAGCCAAGCAGGAAAAGACATGTCAGGGACCTGGTATAGCCAAATT
CTGGGAGACACCCCAGAGGCCAGGGGTGCAGGTGTGAGTTCAGAAATGCATGTTGGTCAGGAATTCTTAGTAACTGATGCCAG
GGGTCAGCCCTCTCCTGCTTAGAGTGAATGTGTGTCCATCCCCATAGAGGTCAGTGTCTTCTACTGGGAGTGGACCTGTGTTG

TCCCCTTAGAGTTCAGCCTCTCCTGCCTGGAGTGACCATATGTCCATCTAAGTGGACATGTTTCCATCTGAGCGGATGTCTGT
CCATCCCCTTAGAGGTCAGCATCTTCTAATTGGAGTAGCCCTGTGTTGTCCTGTTGTCCCCTTAGAGGCCAGCCTCTCCGCCT
GGAGTGACCATATGTCTATCTCAGTGGACGTGTGCCCATCCTCTCAGAGGCCCTCCACCTCCTGTCTGGACCAGACATGGGTG
TCCCTGGATTGAAGACCCCCCTGTCTCTACCACTGACCCCACCACCACCTTCATGTGGGCCATCGTGTACACCATGAGGCCAG
AGTGCAGCTTCCACTCGGTCCCCTCTTATATGTGCACCATCTGCAGGTTCTCAGGCCAACATCCACGTGCTTTGTGATGCTCA
GCAGCCCCACTCAGGGGCTGCGCATGCAATGAGGACGCCCAACTGAGGGCCACATCAGCCACTGTGAAATCAGCTGTGTTGGT
ACTGCTGGGGATGAGGCCCTACTTCTGGGAACAGTGTCTCCATGGTCTCTGGTCATGTGGAGCACAGAGCCTGGGTCCAGACT
GTGGTCTGGAGCGGGTGTTCCTGCAGAGTCCAGCCTCACCCTCTTCTCAGGCAGTCCCTAGGTGGAAGCTCCCTGTGGGTTCA
ACAGCCCAGGATCCAGGACACCCAAGCTGGACGCTT

>*Ammelaphus imberbis*
CAGCAGGGTACATGCACACTGAGGCACCCCAAAACTGGACACAGGAAAAGACGTGTCAGGGACCTGCTATAGCCAAATTCTGG
GAGACACCCCAGAGGCTAGGGGTGCAGGTGTGAGTTCAGAAACGCACGTTGGTCAGGAATTCTGAGTGACTGATGCCAGAGGT
CAGCCCTCTCATGGCTGGAGTGGATGTGTGTCTGTCCCATTAGAGGTCAGTGTCTTTTAGTGGGAGTGGACCTGTGTTGTCCC
CTTAGAAGTCAGCCTCTCCTGCCTGGAGTGACTGTGTGTCCATCTGAGTGGACGTGTGTCCGTCTGAGTGGATGTGTGTCCGT
CCCCTTAGAGGTCAGCATCTTCTAACTGGAGTGAACCTGTGTTGTCCTCTTAGAGGTCAGCCTCTCCTGCCTAGAGTGACTGT
GTGTCCGTCCCCTTAGAGGTCAGTGTCTTCTTACTGGAGTGGACCTGTGTTGTCACCTTAGAGGTCAGCCTCTCCTGCCTGGA
GTGACCATATGTCCATCTGAGTGGACGTGTGCCCATCCCCTCAGAGGCCCTCCCCCTCCTGCCTGCACCAGACGTGGGTGTCC
CTGGCTGGCGGACCCCTCTGTCTGTACCCATGCCCCCACCCCCACCTCCACGAGGGCCATCGTGCACACCATGAGGCCAGAGT
GCAGCTTCCTCTCCGTGCCCTCCTCCATGTGCGCCATCTGCAGGTCCTCACGCCAGCATCCAGGTGCTTCGTGATGCTCAGCA
CCTGTACACAGAGGTCGGTCATCTGTGCTGGGCACAAAGGGCGGCCCGCCACTGAGGGGTGGGGCATGCACTAAGGATGCCCA
ACTGAGGGCCACATTGGCCACTGTGAAATCAACCATGTTGGTACTGCTGGGACTGAGGCACTGCTTCTGGGAACAGGGTCCCC
AGGGTCACTGGTCACATAGAGCACAGAGCCCGGGTCGAGATTGTGGTCTTGGGGAGGTGTTCCTGCAGAGTCCAGCCTCATGC
TCCTTCTGGCAGTCCCTGGGTGGAAGCTCCCTGTGGGTTCAATGCCAAGGAACCACGACACCCAAGCTGGACGCTT

>*Bubalus bubalis*
CAGCAGGGTACATGCACACTGAGGCTCAGGACAGAACCTCCAGACCCCAAGGGTCTTTAGGTTCTGTGGTGGGGGCAATGGGG
GCCGAGGCCAGTGTGAGACTTTAAGTGACTTGGCCAGCAGCCGAGAGAATGGACTGGACACCCCAAAACTGGACACAGGGAAA
GACGTGTCAGGGACCTGGTATAGCCAAATTCTGGGAGACACCCCAGAGGCCAGGGGTGCAGGTGTGCGTTCAGAAATGCGTGT
TGGTTGGGAATTCTGAGTGACTGATGCCAGAGGTTCTCCCTCTCATGCCTGGAGTGGATGTGTGTCCGTCCCATTAGAGGTCA
GTGTCTTCTAATGGGAGTGGACCTGTGTTGTCCTCTTAGAGGTCAGCCTCTCCTGTCTGGCGTGACTGTGTGTCCATCTGAGT
GGACGTGTGCCCATCCCCTCAGAGGCCCTCCCCCTCCTGCCTGGACCAGACGTGGTGTCCCTGGCTGGCGGACCCCCCCGTCC
GTAACCCTGCCCCCACCCCCACCTCCATGAGGGCCGTCATGTACACCATGTGGCCAGAGTGCAGGTTCCTGTCAGTGCCCTCC
TCCATGTGCGCCAACTGCAGGTCCTCACACCAGCATCCAGGTGCTTCGTGATGCTCAGAACCTGCACACAGAGGTCAGTCATC
TGTGCTGGGCACAGAGGGCAGCCCCACTCAGGGGCTGGGCATGCACTGAGGATGCCCAACTGAGGGCCACATTGGCCACTGCG
AAATCAACCGTGTTGGTGCTGCTGGGGTTGAGGCACTGCTTCTGGGAACAGGGTCCCCAGAGTCAGTGGTCATGTGGAGCACA
GAGCCCGGGTCAAGATTGTGGTCTTGGGGGGGTGTTCCTGCAGAGTCCAGCCTCACACTCCTTCAGGCAGTCCCTGGGTGGAA
GCTCCCTGTGGGTTCAATGCCAAGGCTCCAGTGGGTTCAAAGCCAAGGATCCACGACACCCAAGCTGGACGCTT

>*Cervus elaphus*
CAGCAGGGTACATGCACACTGAGGCCCAGGACGGAACCTCCAGACCCCAAGGGTCGTTAGGTTCTGTGGTGGGGGCACCGGGG
TCCGAGGCCAGAGTGAAACATTAAGTGACTTGGCCAGCAGCTGAGAGAATGGACTGGACACCCCAAAACTGGACACAGGAAAA
GACATAGCAGGGTCTGGTTTAGCCAAATTCTGGGAGACACCCCAGAGGCCAGGGTTACAGGTGTGAGTTCAGAAATGCATGTT
GGTCGGGAATTCTGAGTGACTGATGCCAGAGGTCAGCCCTCTCCCACCTGGAGTGGACCTGTGTTGTCCCCTTAAAGGTCAGC
CTCTCCTGCCTGGAGTGACTGTGTGTCCATCTGAGTGGACGTGTGTCCATCTGAGTGGATGAGTGTCCATTCCCTTAGAGGTC
AGCATCTTCTAACTGGAGTGAACCTGTGTTGTTCCCTTAGAGGTCTGCCTCTCCTGCCTGGAGTGACCATATGTCCATCTGAG
TGGACATGTGCCCATCCCCTCAGAGGCCCACCCCTCCTGCTTGGACCTGACGTGGGTGTCCCTGACTGGCAGACCCCCTGCCC
CCGCCCCAACCTCCATGAGGGCCGTCGTGTACACCATGTGGCCAGAGTGCAGGTTCCTCTTGGCGCCCTCCTCTGTGTGCGCC
ATCCGAAGGTCCTCACGCGGGCATCCAGCTGCTTCGTGATGCTCAGCACCTGCACACAGAGGTTGGTTACCTGTGCTGGGCAC
AGAACGCAGCCCCCTGCTCAGGCCTGGGCATGTACTGAGGACGCCCAACTGAGGGCCACATTGTCCACTGTGAAATCAACAGT
GTTGGTGCTGCTGGGGTTGAAGCCCTGCTTCTGGGAACAGGGTCTCCTGGGTCACTGGTGACGTGGAGCACAGAGCCCGGGTC
CAGATTGTGGTCTTGGGGGGCTGTTCCTGCAGAGTCCAGCCTCACCCTCTTCTCAGGCAGTCCCTGGGTGGAAGCGCCCTGTG
GGTTCAACAGCCTGGGCTCCACGACACCCAAGCTGGACGCTT
