# Supplementary material for: X Chromosome-Specific Repeats in Non-Domestic Bovidae
Source: Genes (Basel). 2024 Jan 25;15(2):159. doi: 10.3390/genes15020159 (PMC10887555; doi:10.3390/genes15020159)
Supplement: Supplementary file 1 [file genes-15-00159-s001.zip › Supplementary Table S3.pdf]

Supplementary Table S3. Distribution of BLASTN matches for KDEXr, BTAXr, and ACEXr sequences on X chromosomes of *B. taurus*, *C. hircus*, and *O. aries*

Bos taurus breed Hereford chromosome X, Bos_taurus_UMD_3.1, whole genome shotgun sequence AC_000187

1
↓
↓

25 446 194	25 444 157
28 950 348	28 952 028
28 987 413	28 989 340
29 046 203	29 048 254
35 658 646	35 656 603
35 883 860	35 882 453
36 593 380	36 591 338
37 051 125	37 049 630

↑
↓

140 108 968	140 108 029
142 699 280	142 698 479
143 019 298	143 018 370
143 058 174	143 057 073
143 385 095	143 384 002
143 499 619	143 498 678
143 566 049	143 565 115
143 619 170	143 618 226
143 659 275	143 658 124
143 717 121	143 716 177
143 721 182	143 720 249

↑
↑
148 823 899 bp

Capra hircus breed Yunnan black goat chromosome X, CHIR_1.0, whole genome shotgun sequence NC_022322

1
↓
↓

47 354 576	47 356 617
47 504 084	47 502 041
47 565 313	47 563 234
47 709 634	47 70 7931
47 645 206	47 643 149
**51 053 374**	**51 054 330**
54 370 423	54 368 663
57 592 840	57 590 830
57 632 081	57 630 984

↑
↓

**120 663 121**	**120 664 064**
**121 364 282**	**121 363 334**
**121 368 325**	**121 367 384**
**121 429 633**	**121 428 677**
121 562 035	121 559 939
121 643 548	121 642 467
**121 672 325**	**121 673 139**
**121 950 994**	**121 951 929**

↑
↑
121 952 644 bp

Ovis aries breed Texel chromosome X, Oar_v3.1, whole genome shotgun sequence NC_019484

1
↓
↓

7 052 793	7 053 727
7 077 517	7 076 583
7 081 225	7 080 292
7 361 185	7 360 376
7 390 904	7 391 984
7 432 481	7 431 705
7 475 064	7 476 681
7 569 839	7 570 788
7 632 187	7 633 128
7 636 490	7 637 431
8 401 184	8 400 250
8 545 563	8 546 507

↑
↓

74 946 674	74 947 775
74 986 334	74 988 348
81 582 017	81 581 065
85 018 313	85 020 360
85 092 391	85 094 454
85 133 515	85 135 558
85 306 691	85 304 650

↑
↑
135 437 088 bp

Legend:
matches for BTAXr sequence
matches for KDEXr sequence
matches for ACEXr sequence
